# Sec61s and Sec62/Sec63 Genes Are Essential for Survival by Regulating the Gut and Cuticle Development in *Locusta migratoria*

**DOI:** 10.3390/insects16060550

**Published:** 2025-05-22

**Authors:** Xiaojian Liu, Mingzhu Ji, Jianzhen Zhang

**Affiliations:** 1 Institute of Applied Biology, Shanxi University, Taiyuan 030006, China; 2 Shanxi Key Laboratory of Nucleic Acid Biopesticides, Shanxi University, Taiyuan 030006, China

**Keywords:** *Locusta migratoria*, Sec61 channel, Sec62/Sec63 complex, intestinal tract, cuticle, RNA interference

## Abstract

In eukaryotes, a lot of proteins are transported across the endoplasmic reticulum by the heterotrimeric Sec61 channel. And post-translational transport needs another Sec62/Sec63 complex. However, functions of these genes are poorly explored in insects. In this study, we first identified five Sec genes, named *Sec61α*, *Sec61β*, *Sec61γ*, *Sec62* and *Sec63*, in *Locusta migratoria*. Quantitative reverse-transcription polymerase chain reaction (RT-qPCR) analysis showed that these five genes were expressed in muti-tissues, including wing pad, leg, foregut, midgut, gastric cecum, hindgut, and highly expressed in the integument. Knockdown of *LmSec61α* and *LmSec61γ* by RNA interference (RNAi) lead to the feeding cessation with a mortality rate of 100%. However, there is only 13.4% of ds*LmSec61β*-injected nymphs died before molting. All nymphs injected with ds*LmSec61α* and ds*LmSec61γ* died before molting with the gut atrophy. Furthermore, hematoxylin–eosin staining indicated that the cells of the midguts and gastric caecum were defective, and the microvilli and peritrophic matrix were destroyed seriously after silencing *LmSec61α* and *LmSec61γ*. Knockdown of *LmSec62* and *LmSec63* resulted in high mortality before and during molting. The hematoxylin–eosin (HE) staining and transmission electron microscopy (TEM) results showed that both the formation of the new cuticle and the degradation of the old cuticle were inhibited in ds*LmSec63*-injected insects compared to the controls. Especially, there was no obvious plaques on microvillar tips of the epidermal cells after silencing of *LmSec63*. These results revealed that *Sec61s* and *Sec62*/*Sec63* genes are required in the gut and cuticle development of locusts. Therefore, these genes are potential targets for the control of locusts.

## Simple Summary

Sec61 channel and Sec62/Sec63 complex play key roles in the protein translocation. In this study, *Sec61α*, *Sec61β*, *Sec61γ*, *Sec62* and *Sec63* were identified in *Locusta migratoria*. Then, the functions of these genes were explored using RNA interference. Silencing *LmSec61α* and *LmSec61γ* lead to the feeding cessation, with the defective structure of the midguts and gastric caecum. But the simultaneous RNAi of *LmSec62* and *LmSec63* disrupted locusts molting by regulating the cuticle formation in the nymphal stages.

## 1. Introduction

In eukaryotic organisms, approximately 30% of the proteins are directed to the endoplasmic reticulum (ER) for localization in subcellular organelles [1]. The translocation of nascent precursor polypeptides into the ER is mediated by a Sec61 channel [2]. The Sec61 channel is a heterotrimer membrane protein complex, containing three subunits Sec61α, Sec61β and Sec61γ [3]. As there are some mechanistic differences depending on the precursor protein being translocated during or after its synthesis at the ribosome, there are the co-translational and the post-translational protein translocation mechanisms. The post-translational translocation is enabled by another Sec62/Sec63 complex [4]. More recently, the crystal structure of the Sec complex was obtained by the cryo-electron microscopy (cryo-EM) techniques, which shows how the Sec62/Sec63 activates the Sec61 channel for post-translational protein translocation [5,6,7].

In insects, studies on the Sec complex mainly focus on *Drosophila melanogaster*. It was demonstrated that the Sec complex plays important roles in the development of multicellular tissues. For example, mutations of *DSec61β* were embryonic lethal with severe defects in the deposition of cuticle proteins. Eggs derived from *DSec61β* germ line clones showed defects in dorsal–ventral patterning. Mutant clones of *DSec61β* in adult flies resulted in morphological defects of the eye and leg [8]. The mutant allele of *DSec61β* blocked the traffic of Gurken (the ligand for the Epidermal Growth Factor (EGF) receptor) to the plasma membrane in the oocyte [9]. An eye specific RNA interference (RNAi) screen combined with the Gal4/UAS system revealed that *DSec61β* is essential for the formation of nanoscale protrusion on corneal lens [10]. Ectopic expression of *DSec61α* resulted in the death of neural cell, which is associated with ubiquitinated protein accumulation [11]. In *Bombyx mori*, co-immunoprecipitation experiments indicated that Sec61 promoted the replication of nucleopolyhedrovirus (BmNPV) in BmN cells and Dazao silkworm [12].

*Locusta migratoria* is a destructive agricultural pest due to their polyphagous nature, high food consumption and fecundity [13,14]. RNAi not only makes significant contributions in our understanding of insect biology, but also shows great potential for pest control. The identification of key genes essential for survival of insects is a crucial factor for RNAi-based controlling pests [15]. The translocation of proteins into the endoplasmic reticulum is a conserved pathway in eukaryotes. Therefore, it is imperative to investigate whether *Sec61s* and *Sec62*/*Sec63* genes has an impact on the growth and development of insects.

In the present study, *Sec61α*, *Sec61β*, *Sec61γ*, *Sec62* and *Sec63* genes were identified in *L. migratoria*, and their expression was analyzed across multiple tissues and developmental days in nymphal stages. Further RNAi experiments revealed the essential roles of these genes in the gut and cuticle development of *L. migratoria*. Therefore, this work indicated that *Sec61sα* and *Sec62*/*Sec63* genes are potential targets for the control of locusts.

## 2. Materials and Methods

### 2.1. Insects

The eggs of *L. migratoria* were obtained from the locust breeding center (Cangzhou, China) and incubated in an insect incubator room in our laboratory. They were maintained at a constant temperature of 30 ± 2 °C, 40 ± 10% relative humidity (RH), and a photoperiod of 14 h of light and 10 h of darkness. After hatching, the 1st instar nymphs were transferred to clean cages and maintained under the above conditions. Nymphs were fed with fresh wheat seedlings. The injections of dsRNA and subsequent analyses (i.e., phenotypic analysis, the survival rate assay and body weight were under the same conditions).

### 2.2. Identification of LmSec61s and LmSec62/Sec63 and Bioinformatics Analysis

The putative complementary DNA (cDNA) sequences of *Sec61s* and *Sec62*/*Sec63* genes were obtained from the locust transcriptome database (GEZB00000000) [16]. Then, the translation was performed using the ExPASy translation tool (https://web.expasy.org/translate/, accessed on 1 October 2023). The SMART tool (http://smart.embl.de/, accessed on 1 October 2023) was used to predict deduced domains of the individual protein sequences of LmSec61s and LmSec62/Sec63. The prediction of transmembrane regions in the LmSec61s and LmSec62/Sec63 proteins were done using DeepTMHMM version 1.0.24 (https://dtu.biolib.com/DeepTMHMM, accessed on 1 October 2023). The molecular weight and isoelectric point of these proteins were predicted on ExPASy pI/Mw tools (https://web.expasy.org/compute_pi/, accessed on 1 October 2023).

### 2.3. The Spatiotemporal Expression Analysis of LmSec61s and LmSec62/Sec63

Seven tissues (wing pad, leg, foregut, midgut, gastric cecum, hindgut and integument) were collected from day 2 of the 5th instar nymphs to analyze the tissue-specific expression profiles of *LmSec61s* and *LmSec62*/*Sec63*. To analyze the developmental expression profiles of *LmSec61s* and *LmSec62*/*Sec63*, the whole bodies of different developmental days of 5th instar nymphs were collected. There were five biological replicates, and each biological replicate contained four individuals.

Total RNA of the above samples was extracted using RNAiso Plus (TaKaRa, Kyoto, Japan). The quantity of RNAs was determined using a NanoDrop 2000 spectrophotometer (Thermo Fisher, Waltham, MA, USA). Total 1 µg RNA was used to synthesize cDNAs by using HiScript^®^III RT SuperMix for RT-qPCR (+gDNA wiper) Kit (Vazyme, Nanjing, China), following the manufacturer’s protocol. cDNA was diluted 5-fold as templates. The relative expression of *LmSec61s* and *LmSec62*/*Sec63* was determined using reverse-transcription quantitative polymerase chain reaction (RT-qPCR) on the Bio-Rad system (Bio-Rad Laboratories, Hercules, CA, USA). Each RT-qPCR reaction was prepared with 7.5 μL of ChamQ Universal SYBR RT-qPCR Master Mix (Vazyme, Nanjing, China), 0.6 μL of sense and antisense primers (10 μM), 3 μL of cDNA and 3.3 μL of ddH_2_O. RT-qPCR reaction was performed under the conditions at 94 °C for 2 min, followed by 40 cycles of 95 °C for 15 s and 60 °C for 31 s. Melting curve of each reaction system was analyzed for each primer. *Lmβ-actin* was the reference gene [17]. The primers of *LmSec61s* and *LmSec62*/*Sec63* are shown in Table S1. The relative expressions of *LmSec61s* and *LmSec62*/*Sec63* were calculated using the 2^−ΔCt^ method.

### 2.4. Synthesis of Different dsRNAs

Specific forward and reverse primers of *LmSec61s* and *LmSec62*/*Sec63* were designed to synthesize dsRNA (Table S1). The cDNAs of 1-day-old of the 5th instar nymphs were used as templates to generate T7-containing PCR products by using 2 × Taq PCR Master Mix (TIANGEN, Beijing, China), following the manufacturer’s protocol. The PCR products were subcloned using pEASY^®^-T3 Cloning Kit (TIANGEN, Beijing, China) and sequenced via Sanger sequencing. The dsRNAs were obtained using the T7 RiboMAX Express RNAi System (Promega, Madison, WI, USA). The specificity of dsRNAs was determined on a 1.5% agarose gel. The concentrations of dsRNAs were adjusted to 2.0 μg/μL using a NanoDrop 2000 spectrophotometer (Thermo Fisher, Waltham, MA, USA).

### 2.5. Body Weight and Survival Rate Assay After LmSec61s RNAi

Ten micrograms of different dsRNAs (ds*GFP*, ds*LmSec61α*, ds*LmSec61β*, ds*LmSec61γ*) were injected into the hemocoel at the abdominal segments of 1-day-old of the 5th instar nymphs (N5D1) using a microsyringe (Gaoge Co., Ltd., Shanghai, China), respectively. The whole bodies of ds*GFP*, ds*LmSec61α*, ds*LmSec61β*, ds*LmSec61*-injected nymphs were collected to analyze the silencing efficiency of each *LmSec61* at 24 h post-injection through RT-qPCR using the same methods described above. Six independent biological replicates were set, and each replicate contained three nymphs. In addition, 30 locusts injected with dsRNAs against each gene were used to analyze the survival rate.

To investigate the effects of gene deletion on the feeding, more than 50 locusts injected with 10 μg of dsRNAs (ds*GFP*, ds*LmSec61α*, ds*LmSec61β*, ds*LmSec61γ*), respectively, were fed with the same weight of wheat seedlings every day to record the food intake. On the 4th feeding day of 5th instar nymphs (N5D4), 12 locusts (six males and six females) were fed 15 g of wheat seedlings; after 24 h, the food consumption of the locusts was analyzed. To compare the changes of body weights post-dsRNA injection, we measured and compared the body weights of locusts at N5D1 and N5D4 using an electronic balance (Sartorius, Göttingen, Germany), respectively. Three locusts were used in one biological replicate, and six biological replicates were set.

### 2.6. The Gut Changes After LmSec61s RNAi

To further explore the changes of guts after *LmSec61s* RNAis, the entire intestines of day 4 of 5th instar nymphs were dissected and imaged with EPSON Perfect V700 photo (Jakarta, Indonesia). The lengths of the foregut, midgut, gastric cecum and hindgut were measured using a Vernier caliper (Guilin Measuring Tools, Guilin, China).

The midguts and gastric cecum were dissected from N5D4 nymphs to further study the effects of *LmSec61s* RNAi on the gut development. The methods of hematoxylin–eosin (HE) staining were the same as described previously [18]. Images of these stained paraffin sections were obtained by an OLYMPUS BX51 microscope (Olympus Corp., Tokyo, Japan).

### 2.7. The Survival Rate and Cuticle Development Analysis After LmSec62/Sec63 RNAi

Ten micrograms of different dsRNAs (ds*GFP*, ds*LmSec62*, ds*LmSec63*, ds*LmSec62* + ds*LmSec63*) were injected into N5D1 nymphs as described previously. After dsRNA injection for 24 h, the whole bodies of ds*GFP*−, ds*LmSec62*−, ds*LmSec63*−, ds*LmSec62* + ds*LmSec63*-injected nymphs were used to analyze the silencing efficiency through RT-qPCR using the same methods described above.

To further study the changes of the cuticle after *LmSec63* RNAi, the integuments of the second abdominal segment of N5D7 nymphs after ds*LmSec63* and ds*GFP* injection were dissected. These microsections and H&E staining were performed following the same methods described above.

### 2.8. Transmission Electron Microscopy (TEM) of the Integument

To further investigate the ultrastructural changes after *LmSec63* silencing, transmission electron microscopy (TEM) was conducted as described previously [19]. In brief, the integuments of the second abdominal segment from day 7 of the 5th instar nymphs that treated with ds*GFP* or ds*LmSec63* were prepared, then sectioned into ultrathin slices and collected onto copper grids. A JEM-1200EX transmission electron microscope (TEM, JEOL, Tokyo, Japan) was used for capturing the images.

### 2.9. Statistical Analysis

The relative expression data of different genes in various tissues and developmental days were analyzed by one-way analysis of variance (ANOVA), followed by Tukey’s test by the SPSS software (version 19.0; SPSS Inc., Chicago, IL, USA). Different letters above the bars indicate significant difference (*p* < 0.05). Two-group comparisons (i.e., silencing efficiency, weight gain, length of guts and cuticle thickness) were evaluated using Student’s *t*-test, and asterisks indicate significant difference (*, *p* < 0.05; **, *p* < 0.01; and ***, *p* < 0.001). All data were showed as mean ± standard deviation (SD).

## 3. Results

### 3.1. Identification of LmSec61s and LmSec62/Sec63 and Bioinformatic Analysis

**Figure 1:**
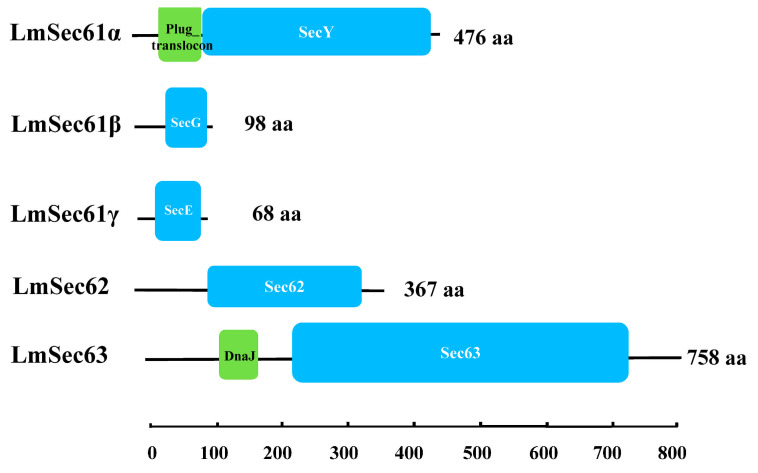
Conserved domains of LmSec61s and LmSec62/Sec63 of *L. migratoria*. Different domains of each Sec protein were shown in distinct colors. “aa” denotes amino acid.

Based on the transcriptomic database, six transcripts encoding Sec61, Sec62 and Sec63 proteins were identified, which were named as *LmSec61α*, *LmSec61β*, *LmSec61γ*, *LmSec62* and *LmSec63* (GenBank accession numbers: PV426902-PV426906). These genes encode proteins of 476, 98, 68, 367 and 758 amino acid residues, respectively. The deduced Mw of LmSec61α, LmSec61β, LmSec61γ, LmSec62 and LmSec63 was 55.33, 10.14, 7.69, 42.77 and 88.05 kDa, respectively. The deduced pI of these proteins was 8.50, 11.08, 9.92, 6.23 and 5.54. Among them, LmSec61α contains a plug domain, SecY domain and 10 transmembrane regions. LmSec61β contains a SecG domain and one transmembrane region. LmSec61γ contains a SecE domain and one transmembrane region. LmSec62 contains a Sec62 domain, while LmSec63 contains a DnaJ domain and a Sec63 domain. LmSec62 and LmSec63 have two and three transmembrane regions, respectively (Figure 1 and Table 1).

### 3.2. Tissue and Developmental Expression Patterns of LmSec61s and LmSec62/Sec63

**Figure 2:**
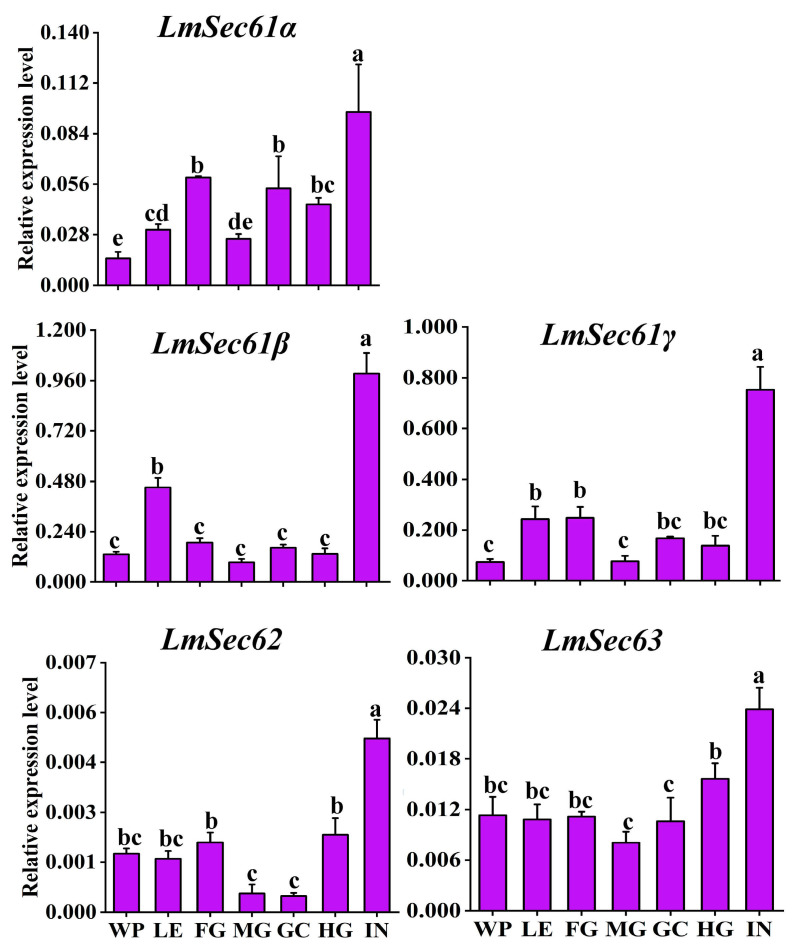
Expression patterns of *LmSec61s* and *LmSec62*/*Sec63* in different tissues of 2-day-old 5th instar nymphs, as revealed by RT-qPCR. WP: wing pad; LE: leg; FG: foregut; MG: midgut; HG: hindgut; GC: gastric cecum; IN: integument. Different letters above the bars indicate significant difference (*p* < 0.05).

The expression levels of *LmSec61s* and *LmSec62*/*Sec63* in different tissues of N5D2 nymphs were examined using RT-qPCR. The expression level of *LmSec61s* were notably higher in the integument, but low in wing pad, leg, foregut, midgut, gastric cecum, hindgut. *LmSec62* were highly expressed in the integument, followed by the wing pad, leg, foregut, and hindgut, and low in midgut and gastric cecum. *LmSec63* was stably expressed in other tissues, abundantly in the integument (Figure 2).

**Figure 3:**
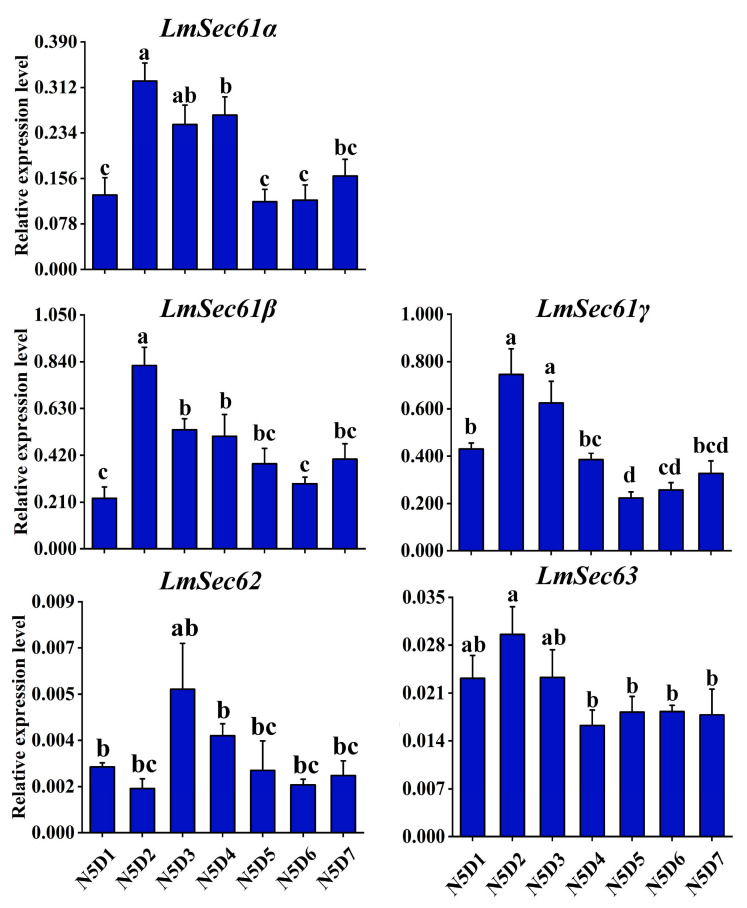
Expression pattern of *LmSec61s* and *LmSec62*/*Sec63* in nymphs during 5th instar stage, as revealed by RT-qPCR. N5D1–N5D7: from day 1 to day 7 of 5th instar nymphs. Different letters above the bars indicate significant difference (*p* < 0.05).

The developmental days of 5th instar nymphs expression profiles of *LmSec61s* and *LmSec62*/*Sec63* were explored using RT-qPCR. *LmSec61α*, *LmSec61β* and *LmSec61γ* were highly expressed in N5D2-N5D4 locusts and had relatively low levels in N5D5-N5D7 locusts. *LmSec62* and *LmSec63* were stably expressed on all seven developmental days (Figure 3).

### 3.3. Effect on Nymphal Survival and Body Weight After LmSec61s RNAis

**Figure 4:**
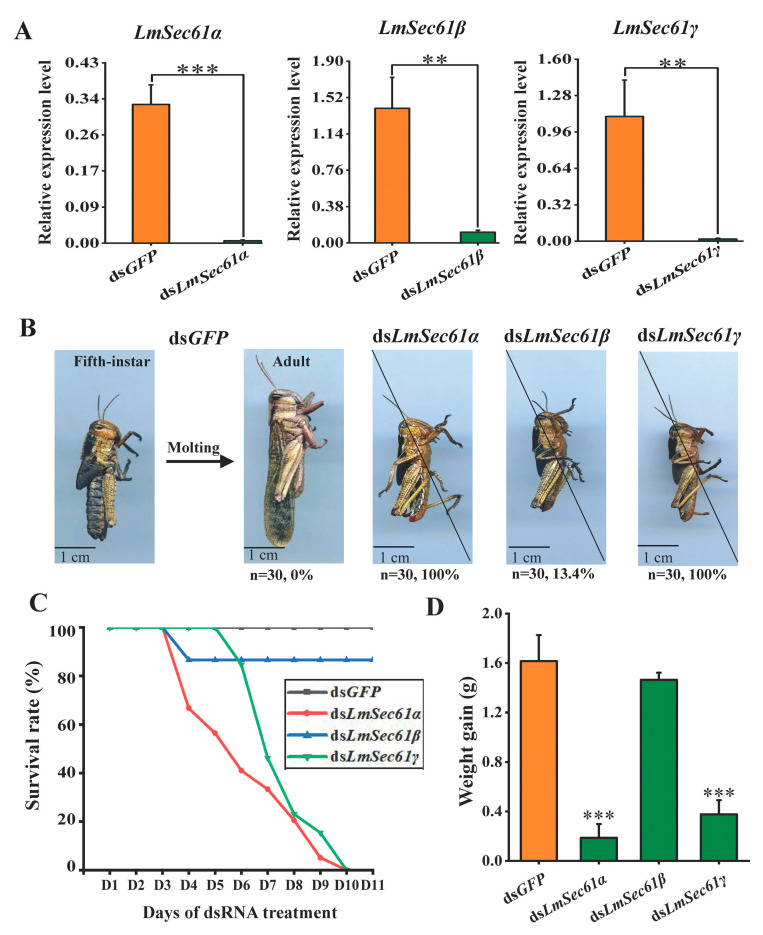
Silencing efficiency and the phenotypes after ds*LmSec61α*, ds*LmSec61β* and ds*LmSec61γ* injection in 5th instar nymphs. (**A**) Relative expression level of *LmSec61α*, *LmSec61β*, and *LmSec61γ* after the ds*GFP*- or ds*LmSec61α*-, ds*LmSec61β*- and *LmSec61γ*-injection for 24 h as detected by RT-qPCR (**, *p* < 0.01, ***, *p* < 0.001, independent sample *t*-tests). (**B**) Phenotypic analysis after knockdown of *LmSec61α*, *LmSec61β* and *LmSec61γ*. Nymphs died before molting after injection of ds*LmSec61α* (100%), ds*LmSec61β* (13.4%) and ds*LmSec61γ* (100%). Percentage represents mortality of locusts after injection of dsRNAs. (**C**) Daily survival rates. All 5th instar nymphs in control group (ds*GFP*-injection) could molt normally to adults after 7 days. In contrast, locusts treated with ds*LmSec61α* and ds*LmSec61γ* could not molt, and died in succession at a mortality rate of 100%. However, only 13.4% of ds*LmSec61β* injected nymphs died before molting; remaining locusts could molt to adults and develop healthily through the stage. (**D**) Average body weight (means ± SD) per three locusts on N5D1 was compared with those on N5D5 after injection of *LmSec61α*, *LmSec61β* and *LmSec61γ*, respectively (***, *p* < 0.001, independent sample *t*-tests).

To investigate the functions of *LmSec61s* in locusts, RNAi experiments against each *LmSec61* gene were performed in N5 nymphs. First, RT-qPCR was used to measure the silencing efficiency. Compared with the controls following the injection of ds*GFP*, the expression of *LmSec61α*, *LmSec61β* and *LmSec61γ* were significantly suppressed by 98.2%, 92.6% and 98.3% at 24 h after treatment with dsRNAs, respectively (Figure 4A). The control N5 nymphs could molt normally to adults after 7 days and develop healthily during the adult stage. In contrast, locusts treated with ds*LmSec61α* and ds*LmSec61γ* could not molt, retained the nymph form and died at a mortality rate of 100%. However, only 13.4% of ds*LmSec61β* injected nymphs died before molting; the remaining locusts molted to adults and developed healthily through the stage (Figure 4B,C).

After 3 days post dsRNA injection of the 5th instar nymphs, it was found that ds*LmSec61α* and ds*LmSec61γ* treatments gradually stopped feeding (Figure S1). Accordingly, the body weight gains of ds*LmSec61α*- and ds*LmSec61γ*-treated nymphs were significantly decreased as compared with the control. However, there was no significantly change post ds*LmSec61β* injection (Figure 4D).

### 3.4. LmSec61α and LmSec61γ Knockdown Resulted in Gut Atrophy

**Figure 5:**
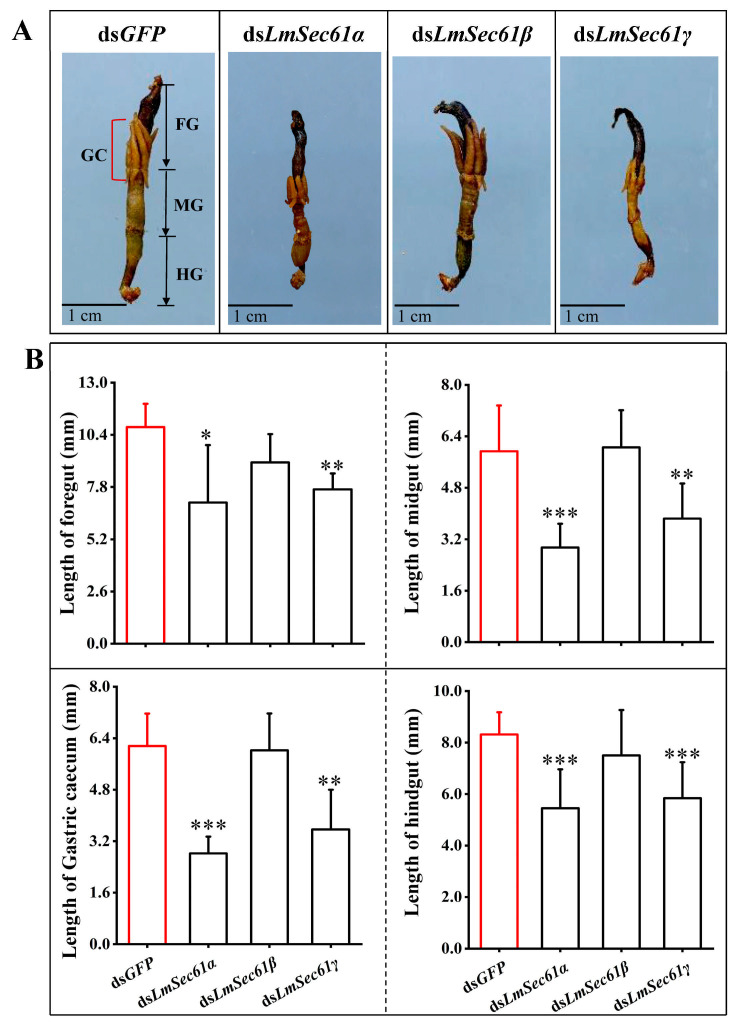
Effects of *LmSec61α*, *LmSec61β* and *LmSec61γ* RNAi on gut development in 5th instar nymphs. (**A**) Guts scanning after *LmSec61α*, *LmSec61β* and *LmSec61γ* RNAis. FG, foregut; GC, gastric caecum; MG, midgut; HG, hindgut. Guts of ds*LmSec61α*- and ds*LmSec61γ*-injected nymphs showed an atrophied phenotype. (**B**) length of foregut, midgut, gastric cecum and hindgut in control and ds*LmSec61α-*, ds*LmSec61β-* or ds*LmSec61γ*-injected nymphs (*, *p* < 0.05, **, *p* < 0.01, ***, *p* < 0.001, independent sample *t*-tests).

To assess the impact after knockdown of *LmSec61s* on the alimentary tract, we dissected and scanned the guts of 5-day-old of dsRNA-treated 5th instar nymphs. As shown in Figure 3, the intestines of the ds*LmSec61α* and ds*LmSec61γ*-depleted locusts were almost empty (Figure 5A) and the guts displayed atrophied phenotypes. The length of the foregut, gastric caecum, midgut and hindgut of ds*LmSec61α* injected nymphs was reduced by 31.92%, 54.24%, 50.39% and 34.50%, while the length of the foregut, gastric caecum, midgut and hindgut of ds*LmSec61γ* injected nymphs were reduced by 28.76%, 40.07%, 37.03% and 29.80% compared to the control (Figure 5B). In contrast, the intestines of the ds*GFP*- and ds*LmSec61β*-depleted locusts were substantially filled with leaf residues (Figure 5A). There was no significant difference in the length of the foregut, gastric caecum, midgut and hindgut between the controls and ds*LmSec61β* injected nymphs (Figure 5B). Therefore, knockdown of *LmSec61α* and *LmSec61γ* impairs the intestinal development of locusts and affected their feeding.

### 3.5. The Histological Changes in the Midguts and Gastric Cecum of dsLmSec61s-Treated Locusts

**Figure 6:**
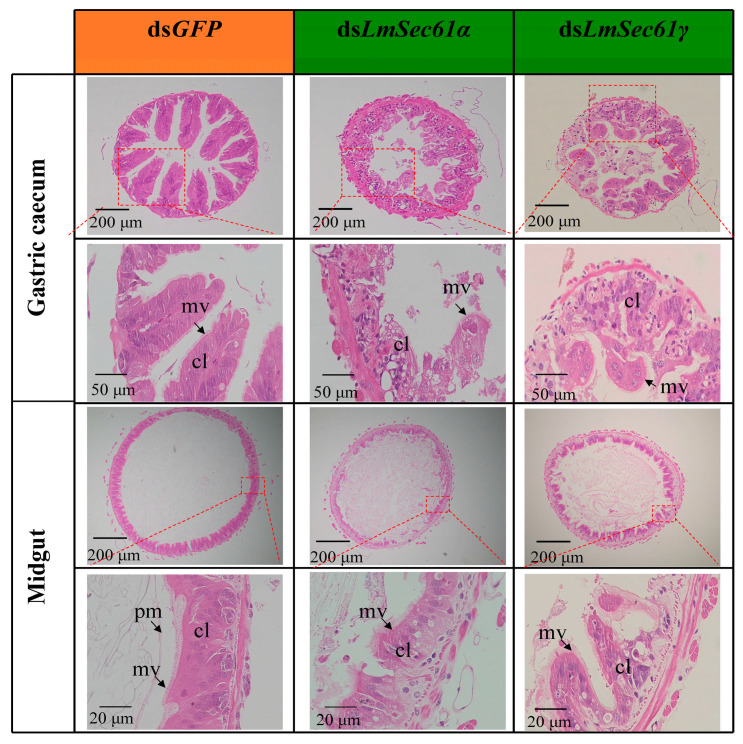
Histological analysis of gastric cecum and midguts after *LmSec61α*, and *LmSec61γ* knockdown. Gastric cecum and midguts were stained with H&E. Structure of columnar cells was damaged in ds*LmSec61α*- and *LmSec61γ*-treated nymphs, and microvilli and peritrophic matrix were defective. cl, cell layer; mv, microvilli; pm, peritrophic matrix.

To further investigate the impact of ds*LmSec61α* and ds*LmSec61γ* injection on microstructural alterations during the gut development, we stained the midguts and gastric caecum of nymphs injected with ds*GFP*, ds*LmSec61α* and ds*LmSec61γ* using H&E. The results showed that the columnar cells were tightly arranged and the microvilli were dense in the controls. In the actively feeding insects, the midguts contained a fully developed peritrophic matrix (PM), which clearly separated the food from the epithelial cells. However, the structural integrity of columnar cells in ds*LmSec61α*- and ds*LmSec61γ*-injected nymphs were damaged, and the microvilli were destroyed seriously. The PM was also significantly disrupted after *LmSec61α* and *LmSec61γ* RNAis (Figure 6).

### 3.6. Effect on Nymphal Survival After Knockdown of LmSec62/Sec63

**Figure 7:**
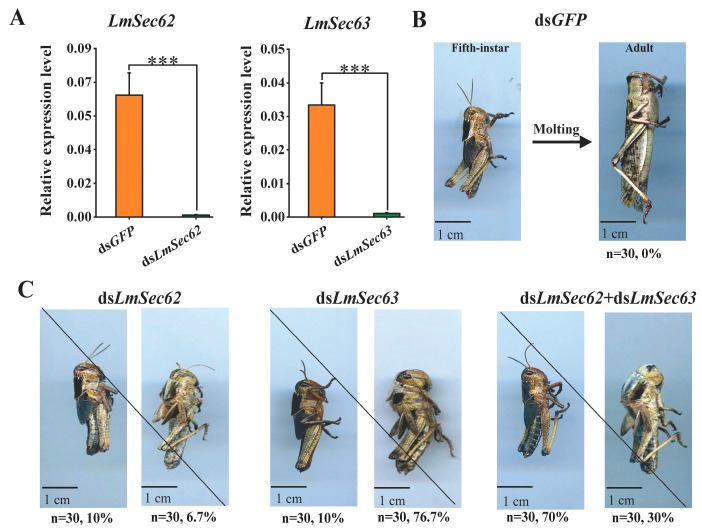
Silencing efficiency and phenotypes after ds*LmSec62* and ds*LmSec63* injection in 5th instar nymphs. (**A**) Relative expression level of *LmSec62* and *LmSec63* after the ds*GFP*- or ds*LmSec62*-, ds*LmSec63*- and ds*LmSec62*+ds*LmSec63* injection for 24 h, as detected by RT-qPCR (***, *p* < 0.001, independent sample *t*-tests). (**B**). All nymphs injected with ds*GFP* could molt normally to adults after 7 days. (**C**) Phenotypic analysis after knockdown of *LmSec62* and *LmSec63*. ds*LmSec62*-, ds*LmSec63*− and ds*LmSec62* + ds*LmSec63*-injected nymphs died before molting and during ecdysis. Accumulative mortality of insects injected with ds*LmSec62* and ds*LmSec63* was 16.7% and 86.7%, respectively. All nymphs injected with ds*LmSec62* + ds*LmSec63* died before and during molting process. Percentage represents mortality of locusts after injection of dsRNAs.

To investigate the functions of *LmSec62* and *LmSec623* in the development of locusts, RNAi experiments against *LmSec62* and *LmSec63* genes were performed in the 5th instar nymphs. The expression of *LmSec62* and *LmSec623* were significantly suppressed by 98.5% and 96.9% at 24 h after treatment with ds*LmSec62* and ds*LmSec63*, as compared with the controls (Figure 7A). All 5th instar nymphs in the control group could molt normally to adults after 7 days (Figure 7B). It was found that ds*LmSec62* and ds*LmSec63* treatments exhibited normal feeding (Figure S1). About 10% of locusts affected by ds*LmSec62* treatments died before molting, and about 6.7% of nymphs died during ecdysis. The other ds*LmSec62*-injected nymphs molted to adults successfully after 7–8 days post dsRNA-injection (Figure 7C). About 10% of locusts affected by ds*LmSec63* treatments died before molting, and 76.7% of nymphs died during ecdysis. The other ds*LmSec63*-injected nymphs molted to adults successfully after 7–8 days post dsRNA-injection (Figure 7C). The accumulative mortality of insects injected with ds*LmSec62* and ds*LmSec63* were 16.7% and 86.7%, respectively. All nymphs injected with ds*LmSec62* + ds*LmSec63* died before and during the molting process.

To confirm the universality of this phenotype induced by d*sLmSec62* and ds*LmSec63*, six micrograms of different dsRNAs (ds*LmSec62*, ds*LmSec623* and ds*LmSec62* + ds*LmSec63*) were injected into 3rd instar nymphs. After dsRNA injection, the same molting barrier was observed after knockdown of *LmSec62* and *LmSec623* (Figure S2).

### 3.7. Effect on the Cuticle Development After Knockdown of LmSec63

**Figure 8:**
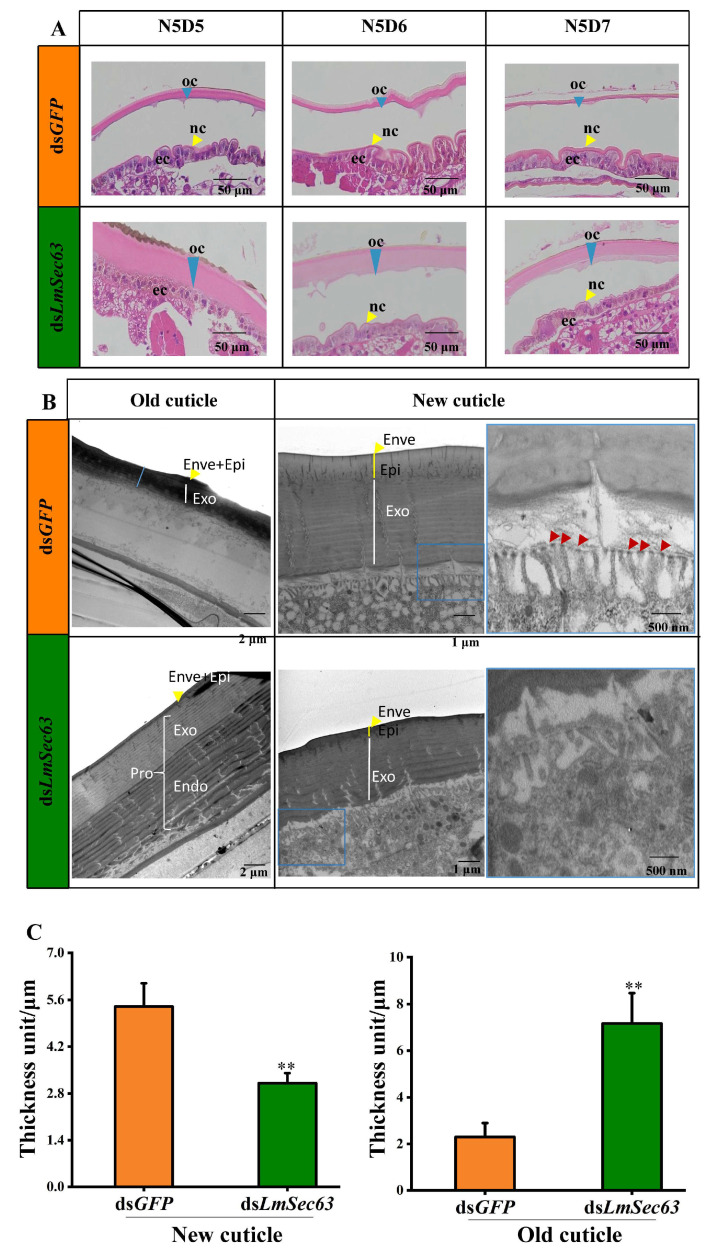
Microscopic sections with H&E and TEM analysis of cuticle after *LmSec63* RNAi. (**A**) H&E staining of integuments. oc, old cuticle; nc, new cuticle; ec: epidermal cell. (**B**) TEM analysis of integuments. Both formation of the new cuticle and degradation of the old cuticle were inhibited in ds*LmSec63*-injected insects compared to controls. Normal microvilli, each containing a single plaque at its tip, were observed in controls, but there were no obvious plaques on microvillar tips of epidermal cells from ds*LmSec63*-injected nymphs. Red arrow indicates plaques on microvillar tips. Enve: envelope; Epi: epicuticle; Pro: procuticle; Exo: exocuticle; Endo: endocuticle. (**C**) Thickness (unit/μm) of cuticles were measured (**, *p* < 0.01, independent sample *t*-tests).

Integument microsections of ds*GFP*- and ds*LmSec63*-injected nymphs were prepared for H&E staining to observe the changes of cuticle. In the ds*GFP*-injected control, the old cuticle successfully separated from the underlying epidermal cells at day 5. The thickness of the new cuticle increased, with the digesting of the old cuticle during the molting process. Compared to the ds*GFP* group, the ds*LmSec63* treatments had a significant negative effect on the cuticle, in which formation of the new cuticles of the ds*LmSec63*-injected nymphs were inhibited than those of the control. Moreover, the old cuticle was thicker after the simultaneous RNAi of ds*LmSec63*. In addition, the detachment of the old cuticle was delayed at day 6 (Figure 8A).

To further visualize the ultrastructural alters in the cuticle after knockdown of *LmSec63*, TEM analysis was performed from the dsRNA-treated insects. The old cuticle was normally degraded in the ds*GFP* group with the thickness of 2.3 μm, whereas the degradation of the old cuticle was inhibited in ds*LmSec63* treated insects with the thickness of 7.2 μm. In addition, the thickness of the new cuticle in control nymphs was 5.4 μm. But the thickness of the new cuticle with multi-lamellar layers was dramatically reduced to 3.1 μm in the ds*LmSec63* groups. Silencing of *LmSec63* has a significant effect on the microvillar tips at the surface of the epidermal cells. Normal microvilli, each containing a single plaque at its tip, were observed from controls, but there were no obvious plaques on the microvillar tips of epidermal cells from ds*LmSec63*-injected nymphs (Figure 8B).

## 4. Discussion

### 4.1. Sec Proteins Are Highly Conserved in Organisms

In the present study, we identified five *Sec* genes (*LmSec61α*, *LmSec61β*, *LmSec61γ*, *LmSec62* and *LmSec63*) in the insect pest *L. migratoria*. These proteins contain several transmembrane domains, which is consistent with the structure of Sec61s and Sec62/Sec63 proteins in other organisms. LmSec61α contain a SecY domain, and LmSec61β and LmSec61γ contain a SecG domain and a SecE domain, respectively, which are the functional homologues in *E. coli* [20]. In addition, Sec61α consists of a plug domain, which was identified the characteristic motif in the luminal/extracellular cavity [21]. LmSec62 contains a Sec62 domain, while LmSec63 contains a Sec63 domain and a DnaJ domain, which was demonstrated to allow the interaction with chaperones that facilitate the unidirectional translocation of precursor proteins through the translocation pore of Sec61 channel in *S. cerevisiae* [22].

### 4.2. LmSec61s Are Required for the Gut Development

The insect intestinal tract contains the foregut, midgut and hindgut. The main function of foregut is for the storage of food and grinding, whereas the hindgut plays roles in the excretion of food residues, the retention of water and ion absorption. The digestive cells of midguts are required for absorbing nutrients and secreting digestive enzymes and components of peritrophic matrix (PM) [23]. The gastric caecum is an elongation of the midgut, which increases intestinal surface area, promoting the efficiency of digestion and absorption [24].

In this study, injection of ds*LmSec61α* and ds*LmSec61γ* successfully reduced the expression of each gene, which negatively affected survival rate and inhibited the growth of *L. migratoria* (Figure 4), suggesting that *LmSec61α* and *LmSec61γ* are possible target genes for RNAi-based pest control of *L. migratoria*. We observed that nymphs injected with ds*LmSec61α* and ds*LmSec61γ* consumed less diet (wheat seedlings) compared to the ds*GFP* control (Figure S1). Consistently, the guts from the ds*LmSec61α* and ds*LmSec61γ*-injected nymphs were almost empty and exhibited atrophy (Figure 5). Histological analysis further showed that the structural integrity of the midguts and gastric caecum in ds*LmSec61α* and ds*LmSec61γ* treatments were damaged, and the microvilli and PM were destroyed seriously (Figure 6).

Several studies have demonstrated that the secretion of proteolytic enzymes (trypsin proteases, carboxypeptidases and aminopeptidases) from intestinal epithelial cells is essential for the development of insects [25,26]. In *Aedes aegypti*, knockdown of a midgut serine protease gene (*AaSPVI*) significantly decreased food digestion, inhibiting nutrient absorption and reproduction [27]. Furthermore, the PM, composed of chitin, proteoglycans and proteins, is continuously synthesized and degraded in response to insect feeding. Chitin chains are synthesized by several enzymes; most important is chitin synthase 2 (CHS2), which is essential for maintaining the physiological function of the PM. In *L. migratoria*, we previously showed that *LmCHS2* RNAi significantly damaged the structure of PM [28]. In addition, deficiency of *LmIIM3*, a member of mucin family belonged to a group of glycosylated macromolecules, resulted in defects of the PM in midguts [29].

It is well known that the Sec61 channel is a conserved and heterotrimeric complex in eukaryotes which mediates proteins transported into the ER. The damaged guts in the *LmSec61α* and *LmSec61γ* RNAi locusts undoubtedly inhibit LmSec61 activities, which may block proteins to transport into the endoplasmic reticulum, thus interfering with uptake of nutrients. As a result, 100% mortality was observed for ds*LmSec61α* and ds*LmSec61γ* treatments.

### 4.3. LmSec62/Sec63 Are Essential for the Cuticle Development

The integument is a multifunctional tissue in insects which defines and maintains the shape of the body, prevents dehydration and protects against predators and environmental stressors [30]. The cuticle is composed of three layers (an envelope, an epicuticle and a procuticle). Neutral lipids, wax esters and proteins are the main components in the envelope. The epicuticle contains mainly of proteins and lipids, while the procuticle contains mainly of chitin and proteins, respectively [31].

In this work, we revealed that *LmSec62* and *LmSec63* have an essential role in the development of the cuticle. Injection of ds*LmSec63* resulted in a reduced thickness of the new exocuticle and blocked the degradation of old cuticle, as compared with the control (Figure 8A). The exocuticle is composed of chitin and proteins. During the molting process, enzymes involved in chitin synthesis and degradation and protease are synthesized within epidermal cells and secreted. Therefore, protein secretion is an essential factor in the formation of the cuticle. The cuticle defects are similar with those deficiency of genes responsible for chitin synthesis and degradation. In *L. migratoria*, we previously showed that *LmUAP1* and *LmCHS1* are responsible for the chitin synthesis of new cuticle, while *LmCHT5* and *LmCHT10* are required for degradation of chitin, respectively [32,33]. Eighty-one cuticular protein genes were identified in *L. migratoria*. Most of the genes were expressed in the integument, pronotum and wings, and differentially expressed at different developmental stages [16]. A cuticle protein ACP7 is transported from epidermal cells to exocuticle during wing. Knockdown of *LmACP7* also showed the similar phenotypes [34]. TEM results further showed that there were no obvious plaques on the microvillar tips of epidermal cells from ds*LmSec63*-injected nymphs (Figure 8B). The microvillar tips were proposed to be the points of secretory activity during the formation of new cuticles [34]. A defect in the secretory activity might explain the thinner new exocuticle layer and thicker old cuticle in ds*LmSec63* treatments.

As there are some mechanistic differences depending on the precursor protein being translocated during or after its synthesis at the ribosome, one can distinguish between the co-translational and the post-translational transport mechanism [2]. During co-translational transport, it relies on the ribosome and Sec61 channel. The co-translational translocation exists in all cells and is mainly used for the translocation of secretory proteins and the integration of most membrane proteins. The Sec62/63 complex is involved in the post-translational translocation [3,4]. In this study, the accumulative mortality of insects injected with ds*LmSec62* and ds*LmSec63* were 16.7% and 86.7%, which indicated that there are other secretory pathway members. The mechanism underlying this inhibitory effect on cuticle development after *LmSec62* and *LmSec63* RNAi requires further investigation.

### 4.4. The Possibility of LmSec61 and LmSec63 in Pest Control

RNAi is a promising technology for potential applications in pest control. However, off-target effects of dsRNAs are considerable obstacles. Previous studies in other insects showed that contiguous sequence matches of ≥21 nt to the target gene are essential for dsRNA to trigger RNAi [35,36]. Recently, dsRNAEngineer (https://dsrna-engineer.cn, accessed on 14 March 2025) provides off-target analysis to ensure its biosafety for non-pests [37]. Here, we analyze the specificity and the off-target potential of dsRNAs of *LmSec61α*, *LmSec61γ* and *LmSec63* using this tool. The results showed that there were no contiguous sequence matches of ≥10 nt nucleotide sequences of *LmSec61α*, *LmSec61γ* and *LmSec63* with those in *Apis mellifera* and *Homo sapiens*. Thus, it is indicated that these genes are possible targets for RNAi-based managing *L. migratoria*.

The low efficiency of RNAi in Locusta via the oral delivery of dsRNA is a major limitation, which preventing laboratory RNAi findings to potential field applications because of dsRNase activity [38]. Recently, various delivery vehicles have been developed to protect dsRNA from premature degradation in *L. migratoria*. For example, it was demonstrated that a nanocarrier (the block copolymer poly(ethylene glycol)-polylysine(thiol) [PEG-PLys(SH)] for the oral administration of dsRNAs is effective [39]. In addition, it was reported that the nanocarrier (star polycation) could effectively deliver dsRNA of *Lmidgf4* (imaginal disc growth factor 4) into the locust [40].

## 5. Conclusions

In this study, Sec61s and Sec62/Sec63 genes were first demonstrated to play important roles in the gut and cuticle development in *L. migratoria*. This work provides not only new biological functions of Sec genes, but also targets for RNAi-based managing *L. migratoria*.

## Figures and Tables

**Table 1:** Molecular characteristics of LmSec61 and LmSec62/Sec63.

Name	CDS (bp)	Amino Acids	Mw (KDa)	pI	Transmembrane Domains (TM) (Position; aa)
LmSec61α	1431	476	55.33	8.50	34–53; 77–96; 118–138; 145–165; 173–193; 241–261; 289–309; 354–375; 421–441; 446–462
LmSec61β	297	98	10.14	11.08	72–92
LmSec61γ	207	68	7.69	9.92	36–61
LmSec62	1104	367	42.77	6.23	193–213; 224–245
LmSec63	2277	758	88.05	5.54	13–35; 74–94; 192–212

“aa” denotes amino acid.

## Data Availability

The original contributions presented in this study are included in the article/Supplementary Materials. Further inquiries can be directed to the corresponding author.

## Supplementary Materials

The following supporting information can be downloaded at: https://www.mdpi.com/article/10.3390/insects16060550/s1, Figure S1: Effects of *LmSec61α*, *LmSec61β* and *LmSec61γ* RNAi on food consumption of 5th instar nymphs.; Figure S2: Silencing efficiency and phenotypes in 3rd instar nymphs after ds*LmSec62* and ds*LmSec63* injection; Table S1: Primer sequences used for RNAi and RT-qRCR in this study.
