# Supplementary material for: Sec61s and Sec62/Sec63 Genes Are Essential for Survival by Regulating the Gut and Cuticle Development in Locusta migratoria
Source: Insects. 2025 May 22;16(6):550. doi: 10.3390/insects16060550 (PMC12192934; doi:10.3390/insects16060550)
Supplement: Supplementary file 1 [file insects-16-00550-s001.zip › insects-3579222-supplementary.pdf]

Table S1. Primer sequences used for RNAi and RT-qRCR in this study.

| Primer name | Sequence (5'-3') | Application | Product size(bp) |
|---|---|---|---|
| ds*LmSec61α*-F | taatacgactcactatagggAGATTGGTGCTGGAGTTT | RNAi | 452 |
| ds*LmSec61α*-R | taatacgactcactatagggGATAATGGGAATGTTTGATG | | |
| ds*LmSec61β*-F | taatacgactcactatagggGCTGGAAGTAGATCACCAACG | | 258 |
| ds*LmSec61β*-R | taatacgactcactatagggGCTTCTTGTGTACTTTCCCCA | | |
| ds*LmSec61γ*-F | taatacgactcactatagggGATTCAATCCGCCTGGTAAA | | 113 |
| ds*LmSec61γ*-R | taatacgactcactatagggACAAAGAAGCCAATGAATCCCA | | |
| ds*LmSec62*-F | taatacgactcactatagggCATCGAGCCAAGAAGATTC | | 463 |
| ds*LmSec62*-R | taatacgactcactatagggCCCACAGCAGACAAAAGAC | | |
| ds*LmSec63*-F | taatacgactcactatagggTGGATTGTATGCTCTTGTT | | 517 |
| ds*LmSec63*-R | taatacgactcactatagggGCAGTTTTCTATTGTCTCA | | |
| ds*GFP*-F | taatacgactcactatagggGACGTAAACGGCCACAAGTT | | 496 |
| ds*GFP*-R | taatacgactcactatagggCGACCACTACCAGCAGAACA | | |
| *qLmSec61α*-F | GCTCGTGCTTACCCAGTT | RT- qPCR | 183 |
| *qLmSec61α*-R | TTGCTTTGCCACATCTTT | | |
| *qLmSec61β*-F | TGTCCCAGTGCTTGTCATGT | | 138 |
| *qLmSec61β*-R | TTGGGTTGGAATTGTTTGTG | | |
| *qLmSec61γ*-F | GGTTTCTGCATCATGGGATT | | 104 |
| ds*LmSec61γ*-R | AACAGATGAGAATGACGATCCA | | |
| *qLmSec62*--F | GGTTTCCTGGCATCTTTCT | | 171 |
| *qLmSec62*--R | TCCCTCATTATCTGCTTTT | | |
| *qLmSec63*-F | AAAAAGTGTTGGAGGGTAG | | 213 |
| *qLmSec63*-R | CAGTGAATGTATAGATGCC | | |
| *β-actin*-F | CGAAGCACAGTCAAAGAGAGGTA | | 156 |
| *β-actin*-R | GCTTCAGTCAAGAGAACAGGATG | | |

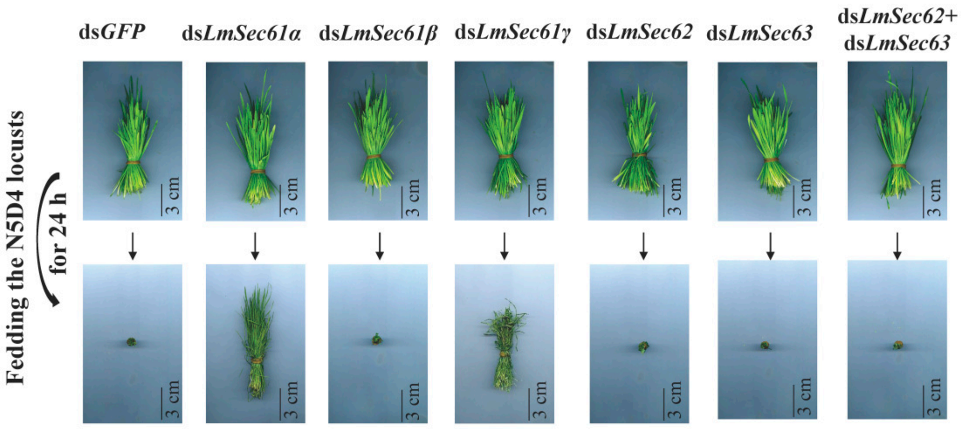


**Figure S1.** Effects of *LmSec61s, LmSec62* and *LmSec63* RNAi on food consumption of 5th-instar nymphs. The dsRNAs of *GFP*, *LmSec61α, LmSec61β, LmSec61γ, LmSec62, LmSec63* and *LmSec62+LmSec63* were injected into newly emerged 5th-instar locusts. On day 4 of the 5th-instar nymphs (N5D4), 6 females and 6 males as a unit group injected with dsRNAs were reared on fresh wheat seedlings. The detached fresh wheat seedlings were imaged again after feeding for 24 h.

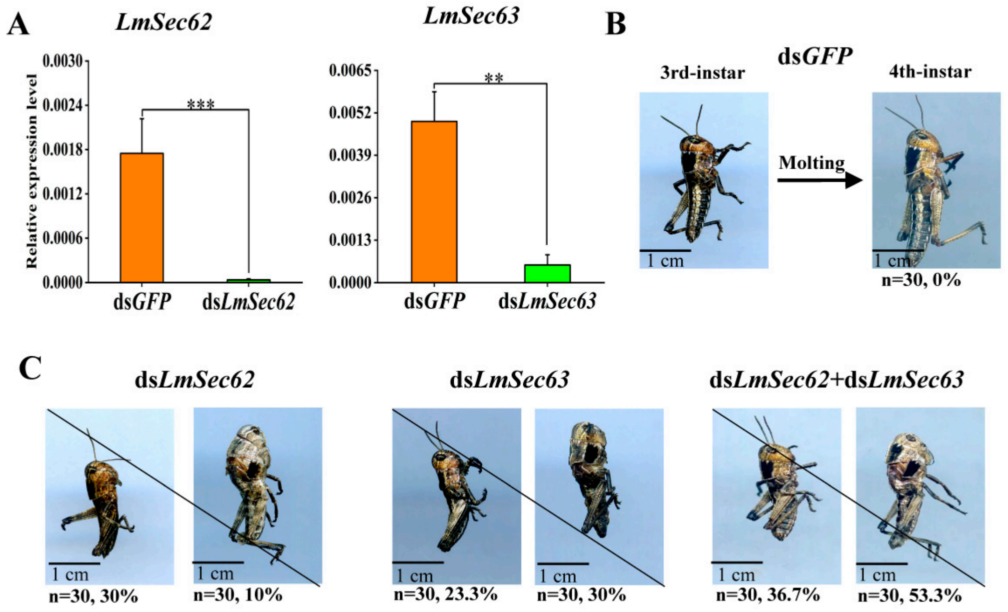


**Figure S2.** Silencing efficiency and the phenotypes in the 3rd-instar nymphs after *LmSec62* and *LmSec63* RNAi. (A) Relative expression level of *LmSec62* and *LmSec63* after the ds*GFP*- or ds*LmSec62*- and ds*LmSec63*-injection for 24 h as detected by RT-qPCR. Data are shown as means ± SD from six independent experiments. ** $P < 0.01$ and *** $P < 0.001$ (independent sample t-tests). (B and C) Phenotypic analysis after knockdown of *LmSec62* and *LmSec63*. The ds*LmSec62*-, ds*LmSec63*- and ds*LmSec62*+ds*LmSec63*-injected nymphs died before molting and during ecdysis. The accumulative mortality of insects injected with ds*LmSec62* and ds*LmSec63* were 40% and 53.3%, respectively. All nymphs injected with ds*LmSec62*+ds*LmSec63* died before and during the molting process. The percentage represents the mortality of locusts after injection of dsRNAs.
